# Skin histopathological responses of *Oreochromis niloticus* (Nile tilapia) to transportation in water with and without salt

**DOI:** 10.1186/s12917-024-03899-y

**Published:** 2024-02-13

**Authors:** Heba Naeim Sadek Hana, Rasha S. A. Abd El-Lateif, Mohamed Abd El Aziz Ahmed Abd El-Galil, Mohamed Abd Allah Mousa, Fatma Abo Zakaib Ali

**Affiliations:** 1 Animal Health Research Institute (ARC), Assiut, Egypt; 2 Fish Diseases and Management Department, Faculty of Veterinary Medicine, Sohag University, Sohag, 82524 Egypt; 3 Animal Nutrition and Clinical Nutrition Department, Faculty of Veterinary Medicine, Sohag University, Sohag, 82524 Egypt; 4 Pathology and Clinical Pathology Department, Faculty of Veterinary Medicine, Sohag University, Sohag, 82524 Egypt

**Keywords:** *O. Niloticus*, Nile tilapia, Transportation, Salt, Stress, Skin, Histopathology, SEM

## Abstract

*Oreochromis niloticu*s (Nile tilapia) is a well-known economic fish species that can thrive under the right environmental circumstances. The transport of live fish, either for food or as companion animals, presents a big issue for animal welfare at the same time it is considered one of stressful conditions. Hence, the present study investigated the skin histopathological responses of *O. niloticus* that were attributed to stress and salt addition during transportation. Three experimental groups of *O. niloticus* the 1st is the control non-transported group (CG), the 2nd is transport in water without salt (PT-S) and the 3rd is transport in water containing 5gL^− 1^salt (PT + S), the last 2 groups were transported in 5 h transport model. Results indicate that the skin of PT-S fish showed a marked decrease in epidermal thickness, decreased number of goblet cells, and an increase in the sub-epidermal and dermal pigments with the presence of large edematous vacuoles. Fish skin from PT + S demonstrated mild hydropic swelling in epidermal cells with normal goblet (mucous) cells density, and more or less normal melanin pigment distribution in sub epidermis and on the dermis layers, however, dermis showed mild edematous spaces. Scanning microscopy of PT-S skin tissue showed few scratched white patches among normal regions that may represent a thickened surface with the decreased number of goblets cell opening, while the PT + S group showed moderate preservation of surface skin architectures with the presence of goblet (mucous) cells opening in spite of presence of slight thickened white patches. The estimated total lesion changes present in PT-S group showed a significant increase (*P* < 0.001) compared with the control (CG) group. On the other hand, PT + S showed significant (*P* < 0.001) improvement in the overall previously recorded changes compared with the PT-S group, and a non- significant change in the histological architectures compared with the control group. Our findings underlined the importance of skin and its mucous cover health during transportation. The use 5 gL^− 1^salt during *O. niloticus* transportation appears to preserve the surface skin features, and keep the goblet (mucous) cells open to the external surface, and may act as a deterrent for the release of mucus from goblet (mucous) cells in response to stress and lessen the stress of transportation.

## Introduction

*O. niloticus* (Nile tilapia) is among the foremost necessary fishes worldwide, with annual global production exceeding 2.6 million metric tons in 2014 [1]. It is one of the best potential species for freshwater aquaculture due to its high-quality meat, high market demand, and well-established rearing technique [2, 3]. One of the aquaculture operations is the fish transportation from one facility to another or during restocking practices, from a hatchery to rivers, lakes or ponds. Transportation is thought to cause stress to fish and ends up in a variety of physiological responses [4].

According to Schulte (2014) [5], stress is a condition in which internal or external factors (stressors) threaten an organism’s ability to maintain homeostasis. As a result, fish attempt to deal with stress by exhibiting a variety of physiological reactions that help them maintain their normal osmolality and homeostasis. Characterization of the response to stress in commercial fish is relevant to enable aquaculture as an economic activity [5]. Furthermore, fish stress was studied to improve fish welfare conditions for reducing mortality and economic losses during and/or after the transportation process.

The fish skin especially the epidermis with its scales and surface mucus provides a protective physical barrier that is important for osmoregulation and pathogen defense. Unfortunately, the skin is susceptible to damage from handling, physical trauma, fighting, predation, environmental irritants and pathogens. These stressors may lead to disruption of skin barrier homeostasis and dysregulation of skin commensals, which may increase stressed fish disease susceptibility. The skin of fish senses and responds to stress factors and it’s equipped with a mucosal immune system called SALT [6] induced in fish environment.

One common practice in freshwater fish facilities that mitigate transport stress is the addition of salt (NaCl) to the water [7, 8]. Salt is cheap and simply applicable in fish farms and plays a great role in the mitigation of stress disturbances particularly osmoregulation, that occur during freshwater fish transportation [9]. The current study aimed to explore the histopathological responses of *O. niloticus* skin to transportation in water with and without sodium chloride (NaCl – salt).

## Materials and methods

### Experimental design and sampling

150 *Oreochromis niloticus* 100 ± 10gs were obtained from a private Tilapia Breeding Farm at Assiut Governorate. Fish were subdivided into 3 groups, 50 fish in each, the 1st group (CG) was used as non-transported control group, the 2nd fish group (PT-S) was transported in water without salt and the 3rd fish group (PT + S) was transported in water containing 5gL^− 1^ NaCl (0.5%). Transport water was obtained from the farm pond. Each transported fish group was transported in a separate tank (180 L capacity) containing 150 L water, the fish were transported for 5 h at stocking density 33.3gL^− 1^ without sedative drug or stopping and with continuous aeration to the Wet Lab of Fish Diseases and Management Department, Faculty of Veterinary Medicine, Sohag University. Just the fish reached to the Wet Lab, they were anesthetized with MS-222 before sampling. Immediately after reaching the lab, skin samples were excised and fixed for histopathology examination.

### Histopathology

#### Light microscopy

##### Haematoxylin and eosin stain (H&E)

For staining with hematoxylin-eosin, skin samples from the examined fish were fixed in 10% neutral buffered formalin. After fixation the specimens were dehydrated in ascending grade (70, 80, 90, and 100%) of ethyl alcohol for one hour in each of the first three concentrations, while two changes of 100% ethyl alcohol were used for half an hour each. The specimens were then cleared in xylene for 20 min. The specimens were then embedded in paraffin wax for one hour and sectioned at 5–7 μm thickness. For staining, sections were deparaffinized by impeding in xylene for 1–24 h. with shaking; and then hydrated in descending grades of ethyl alcohol till 50% and then in distilled water for 5 min for each change. Sections were stained in hematoxylin stain for one minute and then washed in running tap water. The excess of the stain was removed by washing in 0.5-1% hydrochloric acid in 70% ethyl alcohol for a few minutes, and then by distilled water. Subsequently, the sections were stained in eosin for 5 min, after that passed in 95–100% ethyl alcohol, clarified in xylene, mounted by Canada balsam, and then examined microscopically [10].

##### Periodic acid schiff stain (PAS)

Skin samples from the experimental groups were stained with Periodic acid-Schiff (PAS stain) at two different pH values (1 and 2.5) in order to reveal the chemical composition of mucosal secretion and visualize different mucins. The staining method employed was a slightly modified version of the alcian blue (AB)/periodic acid-Schiff’ reagent (PAS) technique described by [11]. The skin between the dorsal and ventral fins was removed from one side of the fish and fixed, the fixed samples were washed in running water. The thin sheet of tissue was rinsed in 3% acetic acid at pH 2.5 for 2 min and stained with 1% alcian blue in 3% acetic acid at pH 2.5 for 15 min then washed for 10 min in running water, the stained tissue was immersed for 30 min in 0.3% sodium carbonate, then washed for 10 min in running water, then the tissue was oxidized in 0.5% periodic acid for 5–10 min, then washed for 10 min in running water, and then placed in Schiff’s reagent for 30 min. The reaction was stopped with three 2-mins baths of 0.05 M sodium bisulphite followed by a final 10-min rinse in running water. Specimens were dehydrated, cleared, and mounted in Bioleite (Ohken Co.). Superficial mucous cells in the skin epidermis were stained either blue (AB-positive, PAS-negative), purple (AB-positive, PAS-positive), or deep red (AB-negative, PAS-positive).

#### Scanning electron microscopy

Tissue samples excised from fish groups were rinsed in physiological saline, dipped briefly in 0.1% solution of S-carboxymethyl-L-cysteine to remove mucus following [12], and then fixed in cold (4 °C) 3% glutaraldehyde in 0.1 M sodium cacodylate buffer (pH 7.4) for 4 h. After fixation, the tissue samples were dehydrated, dried using the Critical Point Dryer (E3000 series; Quorum Technologies), attached to stubs, and then coated with gold using Sputter Coater (SC7620; Quorum Technologies) following [13]. Processed samples were examined under a JEOL 5800LV scanning electron microscope.

#### Histopathologic scoring

Each sample was assigned a score based on tissue histopathological examination [14]. The samples were scored semiquantitative, with the assessment based on the visual field inspection of a minimum of 10 sections from each group. Photographs were taken at a magnification of 40 X and tissue alterations were scored according to set criteria: 1, 2, 3, and 4 (absent, mild, moderate, and severe, respectively) [15], Skin tissue sections were analyzed for the following alterations: epidermal cell thickness, vacuolation, the mucosal contents of goblet (mucous) cells stained with PAS were counted under a microscope and scored as blue or magenta, hypodermal cystic dilatation and mucoid degeneration, melanocytes distribution, and degree of deposition. All the analyses were performed by two researchers by recording the nature and extent of the lesion and its frequency of occurrence in randomly selected sites in the skin tissue and the total lesion score was calculated statistically [16].

### Statistical analysis

Data of all measurements from experimental groups were stated as mean ± standard deviation (SD), and they were estimated by the use of GraphPad Prism Version 5 (San Diego, California, USA) analyzing data using one-way ANOVA with Tukey’s post-hoc multiple comparison tests; the statistical significance was considered at *P* < 0.05 [17, 18].

## Results

### Histopathological study

#### Light microscopy

**Fig. 1:**
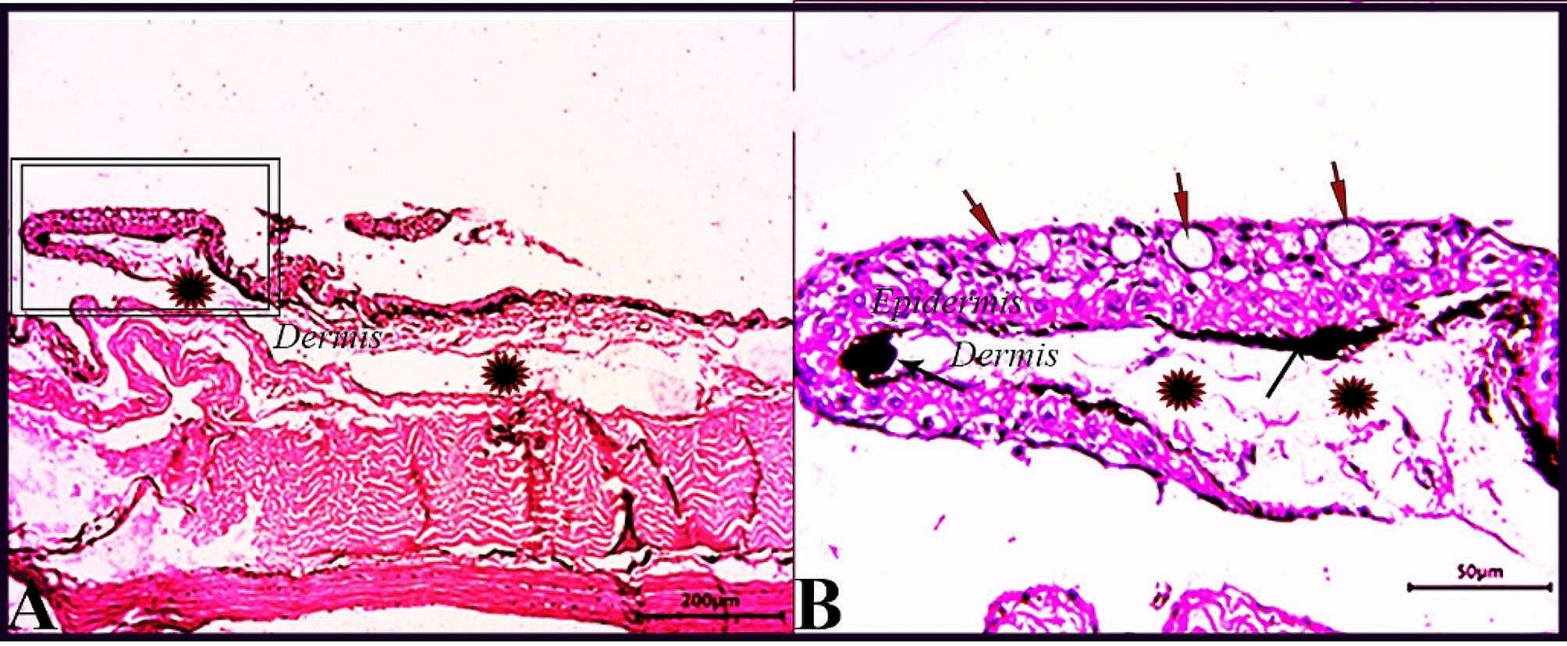
Photomicrograph of skin tissue sections from the *O. niloticus* control fish group **(A magnified in B (A, selected square))** showed normal epidermal covering epithelium; the epidermis was consisted of several layers with dominating goblet (mucous) cells (red arrows), normal pigment aggregations (black arrows), the underlying dermis was free of any inflammatory cells and had loose connective tissue (stars). H&E stain. The bar size was indicated under pictures

**Fig. 2:**
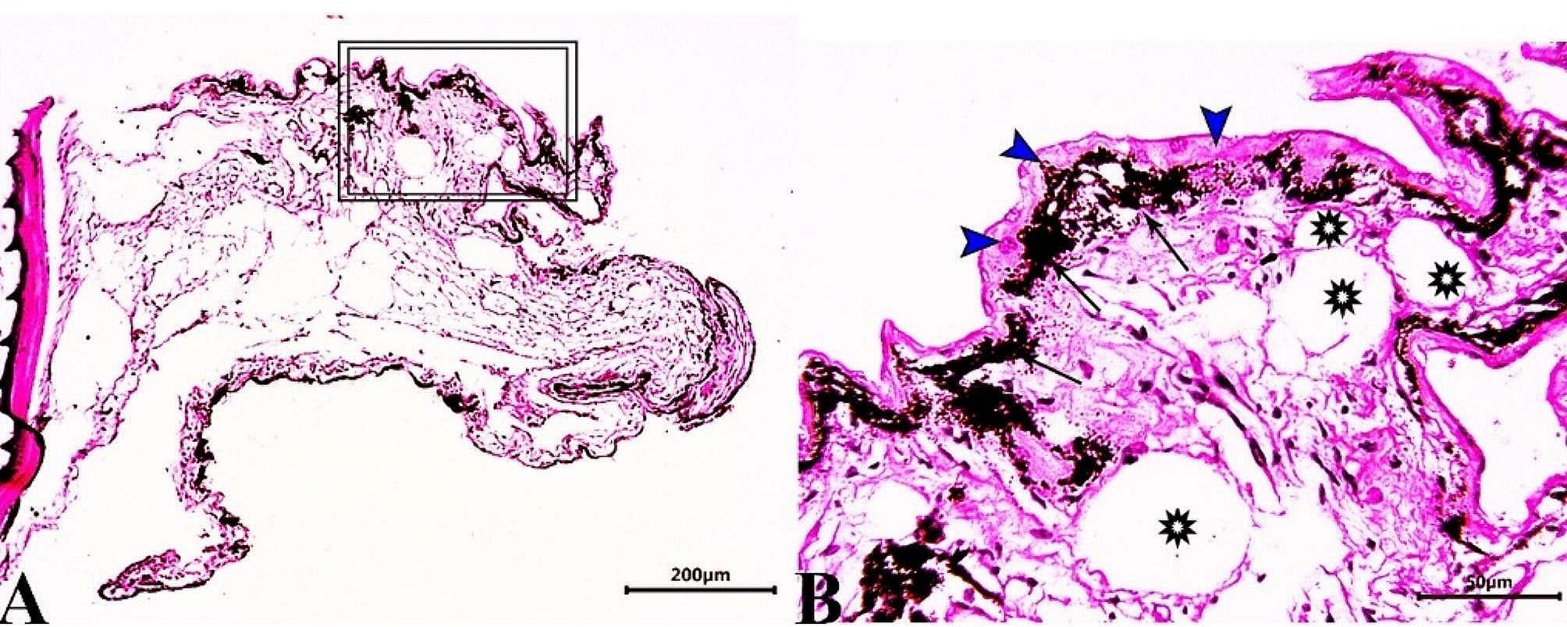
Photomicrograph of skin tissue sections from *O. niloticus* of PT-S group **(A magnified in B (A, selected square))** showed marked thinning epidermal thickness with few scattered goblet (mucous) cells (blue arrowheads), excessive sub epidermal black pigmentation (black arrows) and large edematous vacuoles in the dermis layer (stars). H&E stain. The bar size was indicated under pictures

**Fig. 3:**
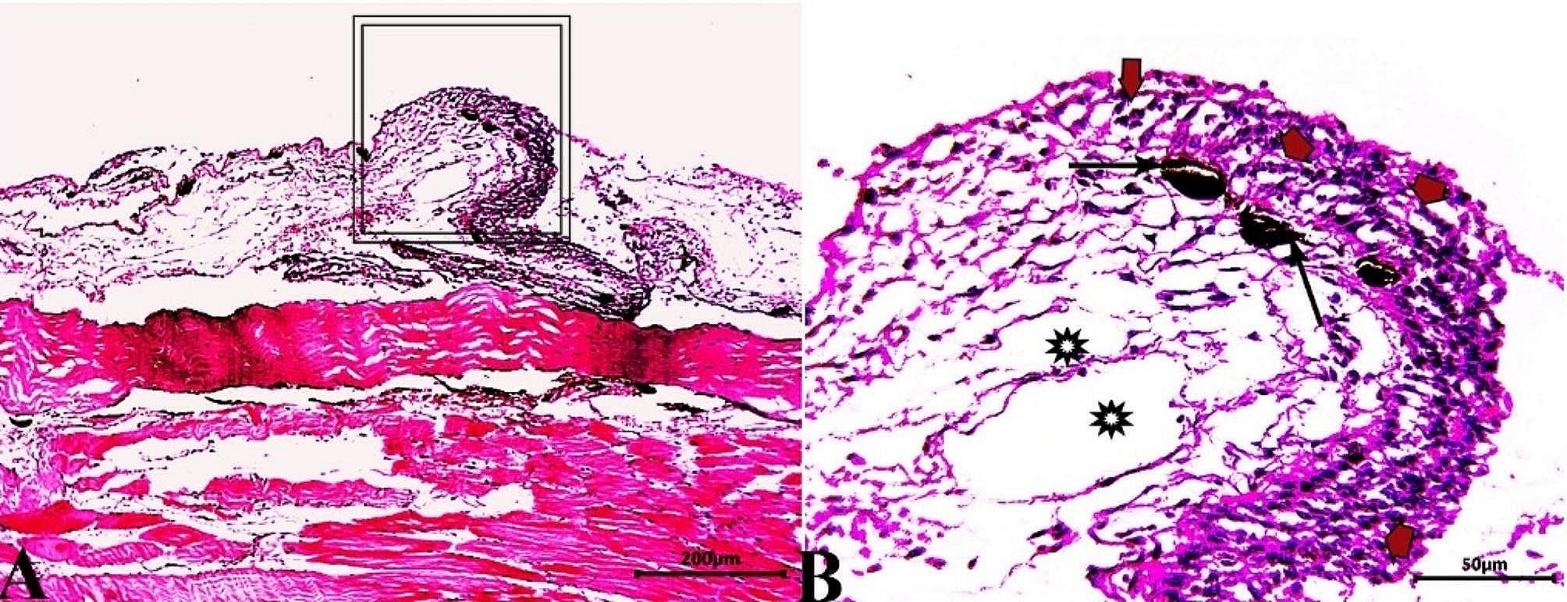
Photomicrograph of skin tissue sections from *O. niloticus* of PT + S group **(A magnified in B (A, selected square))** showed vacuolar degeneration in epidermal cell layers with more or less goblet cell distributed (red arrowheads), mild sub epidermal pigmentation (black arrows) and mild edematous vacuoles in the dermis (stars). H&E stain. The bar size was indicated under pictures

Skin sections from the control group (CG) stained with H & E stain demonstrated normal histological characteristics of the epidermis and dermis, both of which were composed of multiple layers with dominant goblet (mucous) cells and an underlying dermis free of any inflammatory cells and displayed loose connective tissue, on the basal layer of the epidermis, normal levels of pigment aggregations were seen. (Fig. 1). Skin sections from *O. niloticus* (PT-S group) transported in water without salt revealed a considerable decrease in epidermal thickness, decreased number of goblet cells, increased sub epidermal black pigmentation, and large edematous vacuoles were seen in the dermis layer. (Fig. 2). While, skin sections from *O. niloticus* transported in water containing 5gL^− 1^ salt (PT + S group) showed mild hydropic swelling in epidermal cells with the presence of normal goblet (mucous) cells number and a considerable sub epidermal black pigment aggregation nearly similar to control was observed, the dermis still showed edematous spaces (Fig. 3).

**Fig. 4:**
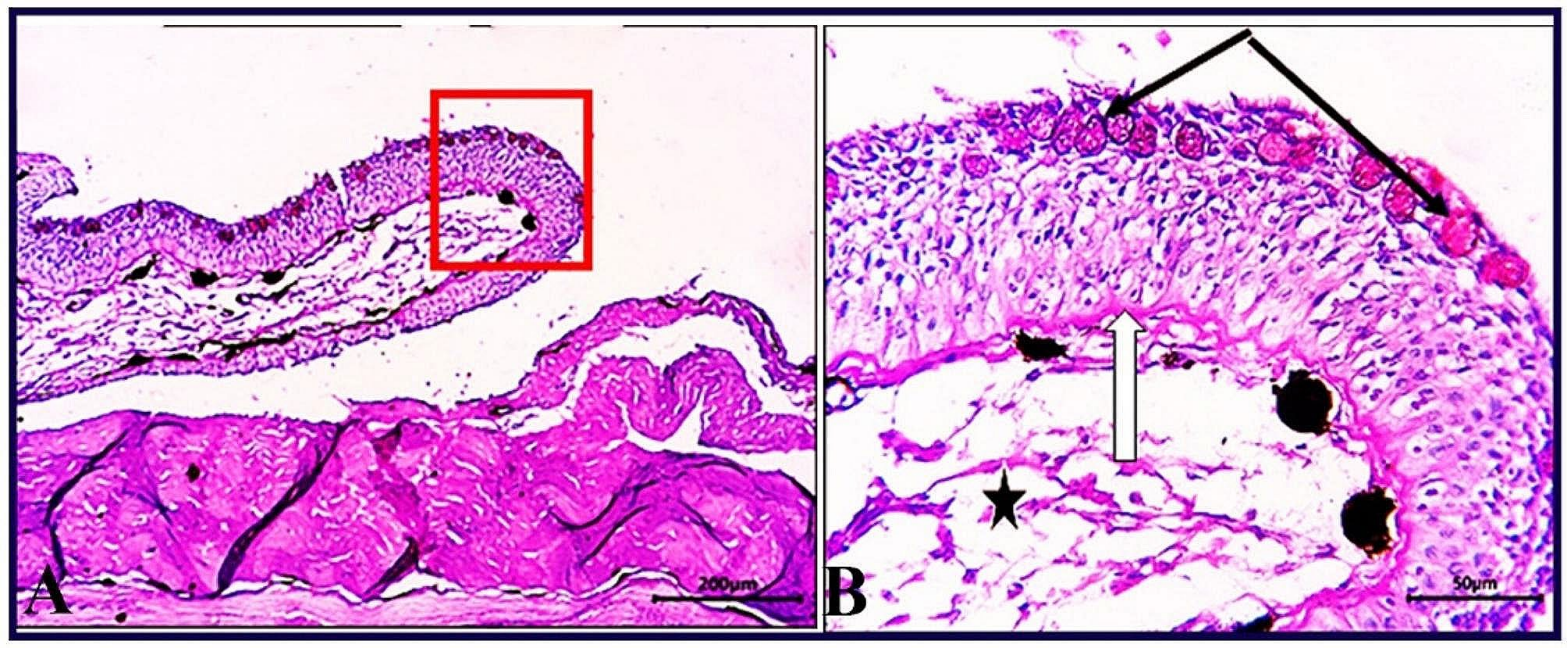
Photomicrograph of skin tissue section from *O. niloticus* control group **(A magnified in B (A, selected square))** showed normal epidermal layer, the dominating goblet (mucous) cells (black arrows), basal lamina of epidermal layer (white arrow), and the fine CT fibers (star) were all stained positively with PAS. The bar size was indicated under pictures

**Fig. 5:**
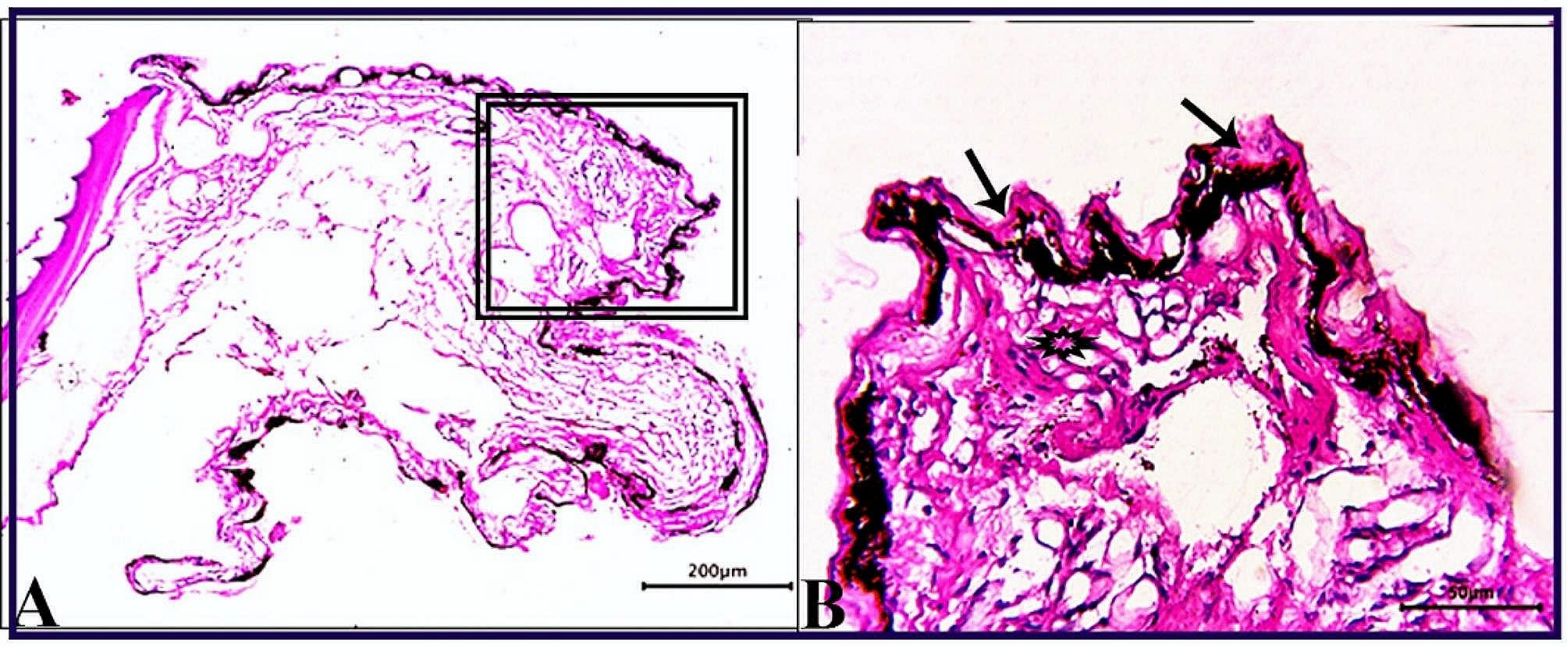
Photomicrograph of skin tissue section from *O. niloticus* of PT-S group **(A magnified in B (A, selected square))** showed thin irregular epidermal cell layer with few goblet (mucous) cells and decrease staining intensity of basal lamina (black arrows), marked connective tissue fibers in dermal layer with increase positivity of PAS reaction (star). PAS stain. The bar size was indicated under pictures

**Fig. 6:**
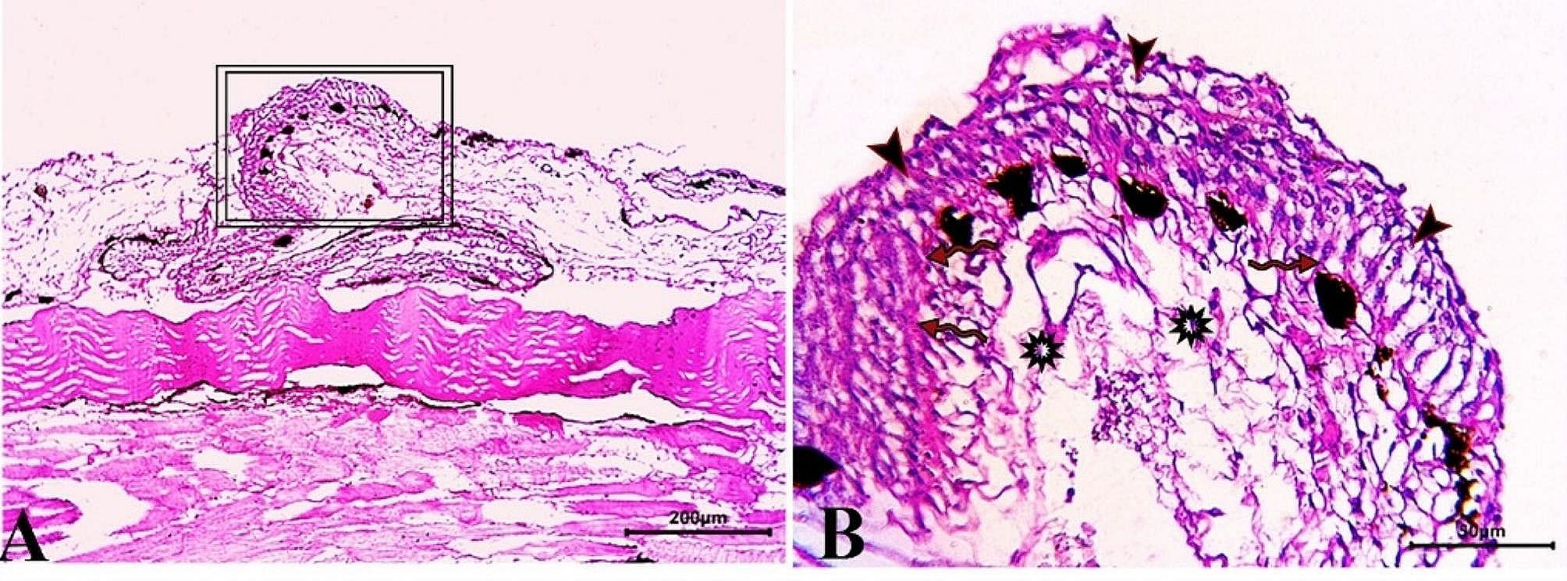
Photomicrograph of skin tissue section of *O. niloticus* of PT + S group **(A magnified in B (A, selected square))** showed normal epidermal cell thickening and organization, except more or less goblet (mucous) cells number (arrowheads), normal epidermal basement (zigzag arrows) and more or less positivity of PAS reaction of dermal connective tissue (stars). PAS stain. The bar size was indicated under pictures

Skin section of *O. niloticus* (CG) stained by PAS showed a reaction reflected the muco-polysaccharides level which demonstrate dominating highly positive goblet (mucous) cells among the epidermal cells, basal lamina of the epidermal layer and the fine CT fibers stained positively with PAS in this group (Fig. 4). In contrast, skin sections of PT-S fish group stained with PAS showed decrease in number of goblet cells, decrease the staining intensity of basal lamina and increase the staining intensity of the connective tissue fibers (Fig. 5). While, the skin sections of PT + S fish group showed improved epidermal cell organization with near normal goblet (mucous) cells number, the basement membrane of epidermis showed PAS-positive staining and the dermal connective tissue showed less PAS-positive reaction compared to PT-S group (Fig. 6).

#### Scanning electron microscopy (SEM)

**Fig. 7:**
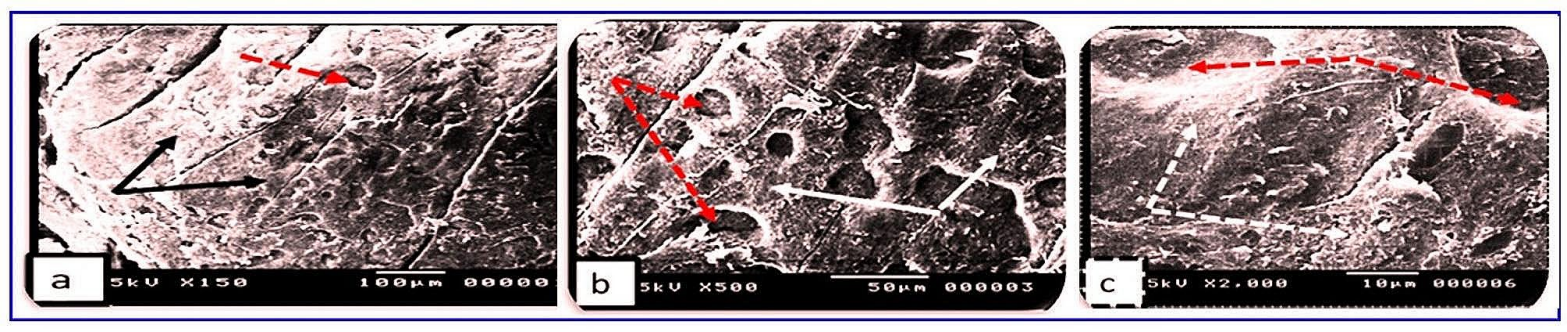
Scanning photographs for skin tissue from *O.niloticus* control group showed (**a**): Regular arranged pattern of surface squamous cells (black arrows) with minor depression indicating the opening of goblet (mucous) cells (red dotted arrows), (**b**&**c**): clearly the opening of goblet (mucous) cells (red dotted arrows) and the flat surface of squamous cells (white arrow). The scale bar was indicated under pictures

**Fig. 8:**
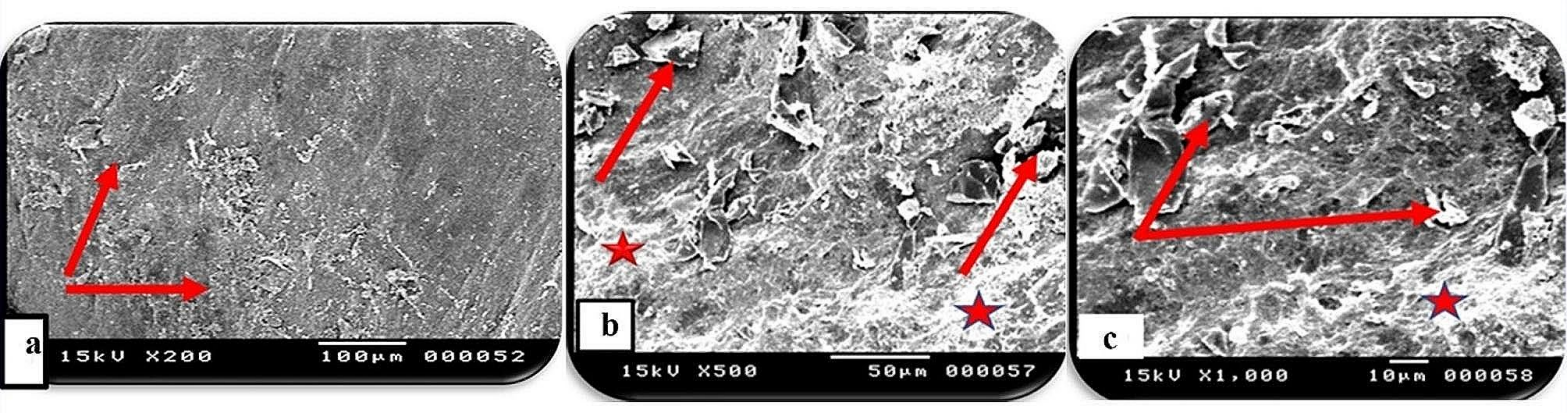
Scanning photographs for skin tissue from *O. niloticus* (PT-S) group showed (**a**): scratched patches (red arrows) among normal regions with scattered observed goblets cells, (**b**&**c**): goblet (mucous) cells opening with cell debris (red arrows) and white patches may represent thickened surface (red stars). The scale bar was indicated under pictures

**Fig. 9:**
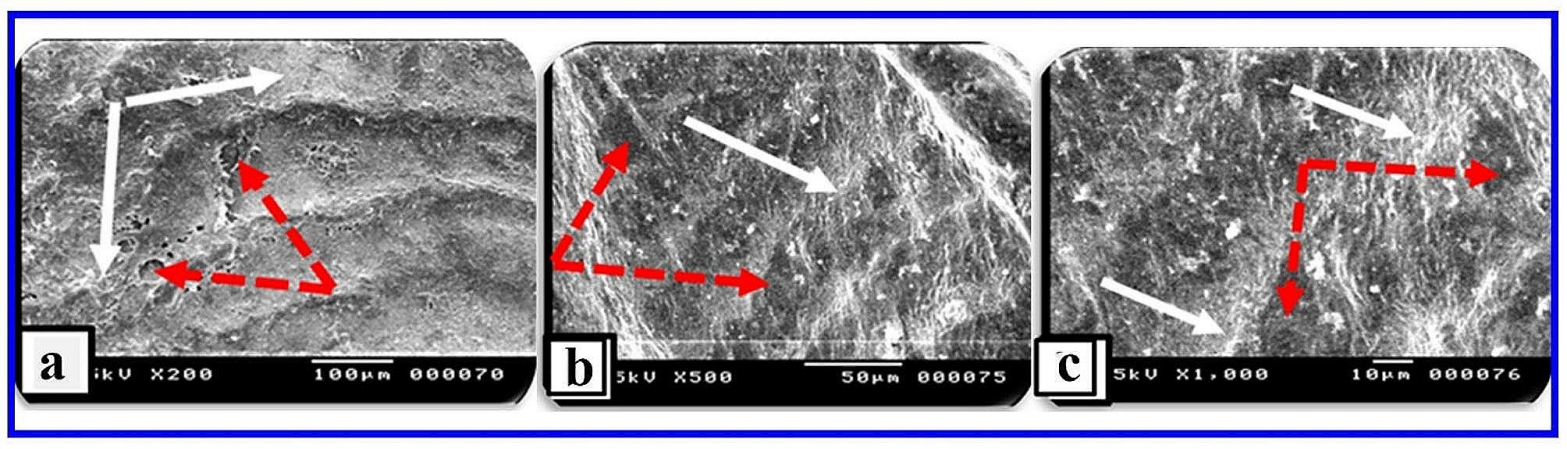
Scanning photographs for skin tissue from *O. niloticus* PT + S group showed (**a**): moderate preservation of surface skin features with some goblet (mucous) cells opening (dotted red arrows) with slight thickened white patches (white arrows), (**b**&**c**): opening of goblet (mucous) cells but not so clear like control (dotted red arrows) separated by slight thickened white patches (white arrows). The scale bar was indicated under pictures

Skin section of the *O. niloticus* control group showed the regularly arranged pattern of surface squamous cells. Minor depressions among superficial cells indicated the opening of goblet (mucous) cells (Fig. 7). In PT-S group, the skin showed few scratched white patches among normal regions that may represent thickened surface with a few numbers of opening of goblets cell (Fig. 8). In the other hand, the skin sections of the PT + S fish group showed moderate preservation of surface skin features with the presence of near normal density and distributed goblet (mucous) cells openings and slight thickened white patches could be observed (Fig. 9). Scanning photos to some extent confirmed what was seen by light microscopy regarding changes occurred in surface epidermal cells.

##### Histopathological scoring

**Fig. 10:**
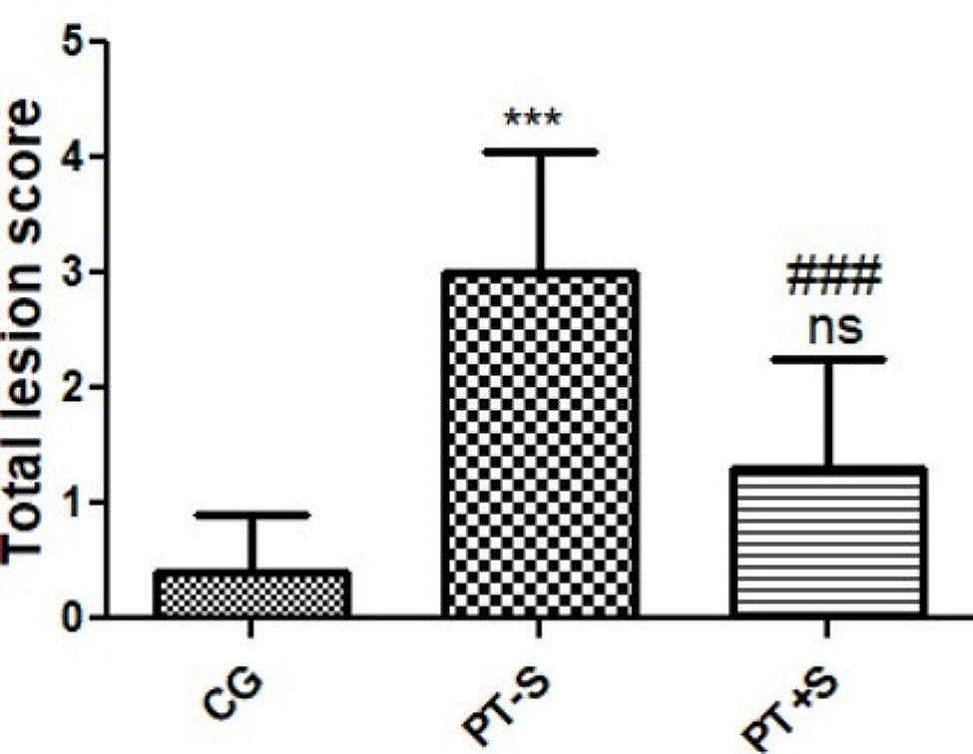
Histomorphometry graph showing semiquantitative measurements of total lesion scores recorded in skin tissue sections among the experimental groups. Data are expressed as means ± standard deviations. Significant differences vs. the control group (CG) are marked by different asterisks, while significant differences vs. PT-S group are marked by different #, all through one-way ANOVA with Tukey’s post hoc test: (***, ###*p* ≤ 0.001). ns means non- significant compared with the CG (control) group

Total lesion changes present in the PT-S group such as epidermal cell thickness, vacuolation, decreased goblet (mucous) cells and their secretions, hypodermal cystic dilatation with mucoid degeneration, and melanin deposition, all showed significant increase (*P* < 0.001) compared with control (CG) group. On the other hand, PT + S showed significant (*P* < 0.001) improvement in the overall previously recorded changes compared with the PT-S group, and a non- significant change in the histological architectures compared with the control group (Fig. 10).

## Discussion

Transportation stress refers to many stress factors that affect the normal physiological state of transported fishes. During transportation, the main cause of stress is the continuous shaking and the mechanical wear caused by the inevitable contact between fish and fish under high-density conditions [19]. The physiological responses of fish to stress are well characterized with regard to endocrine, osmoregulatory, respiratory, and behavioral systems [20]. Moreover, the immune response is generally known to be suppressed in stressed fish [21].

The present work specifically evaluates the changes in *O. niloticus* skin morphology and epithelial barrier integrity following 5 h transportation in water without salt (PT-S) and in water with 0.5% salt (PT + S) and compared with the control non-transported group (CG).

Skin histopathology of *O. niloticus* in the PT-S group showed a marked decrease in epidermal thickness that may be due to injuries sustained during preparation procedures of transportation, the aggressive acts between fish provoked by transport stress causing the loss of protective mucus resulting in an increased risk of injuries and this explanation was supported by [7, 22]. Also, the skin histopathology of *O. niloticus* of this study showed decreased number of goblet (mucous) cells in the PT-S group. Similar findings were consistent by [23] who reported that the number of mucus cells decreased after transport stress, while, our finding wasn’t in line with [24, 25] who found an increased number of mucous cells in the skin in response to external stressors. This may be attributed to the physiological mechanism leading to alteration in the number of skin mucous cells in various stressful conditions is still not clear and this mechanism is not cortisol-related [23]. Previous study suggested that the enumeration of skin goblet (mucous) cells could be used as an indicator of exposure to a stressor where it is well known that a continuous secretion and shedding of mucus produced by skin mucous cells, together with the presence in the mucus of many substances, such as immunoglobulin, complement, lysozyme and lectin protect the fish against various infections [23, 26]. Large edematous vacuoles were seen in the dermis layer of *O. niloticus* in the PT-S group, this observation in dermis may be due to damaging of the epithelial tissue barrier.

The pigment cells in control *O. niloticus* skin (CG) were found at the junction of the epidermis with the dermis. Black pigment cells (melanophores) are often alongside with other pigment cells [27]. Skin pigmentation pattern is a species-specific characteristic that depends on the number and the spatial combination of several types of chromatophores [28]. In PT-S fish, pigmented cells seemed to be different, and the cells became more numerous, larger and disoriented. Similar results were observed by [7]. The morphology of chromatophores was reported to be changed in response to stress. Melanosome dispersion and a darkening of the skin are affected by cortisol [29] so the alteration of pigmented cells could be due to the increased cortisol level observed in *O. niloticus*.

Microscopic examination to the skin sections of the PT + S fish group showed improved epidermal cell organization with a near normal goblet (mucous) cells number, normal epidermal basement and more or less similar to the control dermal connective tissue. These results may be attributed to the fact that the salt has a stress mitigation effect by decreasing the aggressive acts between fish that are provoked by transport stress, acting as a regulator of mucus release from goblet (mucous) cells and reducing the energy requirements for homeostasis and osmoregulation by reducing the difference between the fish and environment osmolality [30].

Scanning electron microscopy revealed differences between the three *O. niloticus* groups. Both transported groups were markedly different from the skin of the control group. In the PT-S group, scratched patches among normal regions on the skin surface were reported, scattered openings of goblet (mucous) cells were observed on the external surface. The results of the PT-S group agreed with previous study [30], who reported that, transport in fresh water caused decreases in mean cellular hemostasis, plasma osmolarity, glycogenesis and hyperglycemia goblet (mucous) cells [31]. Whereas the skin section of the PT + S fish group showed moderate preservation of surface skin features, the goblet (mucous) cells still open to the external surface and released their mucous content during transportation time. The results of the PT + S group in this study suggested that the presence of salt has a stress mitigation effect and may act as a retardant for the release of mucus from goblet (mucous) cells in response to transport-stress, and reduce the need to energy for osmoregulation and maintenance of homeostasis. Our findings come close to previous literatures, documented that, the addition of salt to the water inhibits the decreases in plasma chloride and sodium concentration, which are common consequences of handling stress and also diminishes the hyperglycemia taking place in stress [31]. It was previously recorded that, transport stress may lead to changes in the number of goblet (mucous) cells and amounts of mucus production in the skin of transported animals [25, 32], while, adding salt could mitigate this effect [32, 33]. This finding was supported by previous research suggesting that, addition of salt in transport water reduces the difference between the internal osmolality of fish and that of its environment thereby reducing the physiological workload required to maintain homeostasis [32]. Supporting a link between stress, salinity, and goblet cell biology.

## Conclusion

Our findings have significant importance in the fish aquaculture field and address the importance of skin and its mucous cover health during transportation, we recommend the use of 5gL^− 1^ salt during transportation of *O. niloticus* as the benefits of its use during transport appear to preserve surface skin features and keep the goblet (mucous) cells open to external surface.

## Data Availability

No datasets were generated or analysed during the current study.

## Abbreviations

AB: Alcian blue
H&E: Haematoxylin and Eosin stain
mins: Minutes
*O. niloticus*: *Oreochromis niloticus*
PAS: Periodic acid-Schiff’ Stain
PT + S: Post-transport in water containing 0.5% salt
PT-S: Post-transport in water without salt
SEM: Scanning Electron Microscopy
